# Spatial clustering of livestock Anthrax events associated with agro-ecological zones in Kenya, 1957–2017

**DOI:** 10.1186/s12879-021-05871-9

**Published:** 2021-02-18

**Authors:** Leonard M. Nderitu, John Gachohi, Frederick Otieno, Eddy G. Mogoa, Mathew Muturi, Athman Mwatondo, Eric M. Osoro, Isaac Ngere, Peninah M. Munyua, Harry Oyas, Obadiah Njagi, Eric Lofgren, Thomas Marsh, Marc-Alain Widdowson, Bernard Bett, M. Kariuki Njenga

**Affiliations:** 1 Paul G Allen School for Global Health, Washington State University, Pullman, Washington, USA; 2 Washington State University Global `Health Program-Kenya, WSU, Nairobi, Kenya; 3 School of Public Health, Jomo Kenyatta University of Agriculture and Technology, Nairobi, Kenya; 4 International Livestock Research Institute, Nairobi, Kenya; 5 University of Nairobi, College of Agriculture and Veterinary Sciences, Nairobi, Kenya, University of Nairobi, Nairobi, Kenya; 6 Kenya Zoonotic Disease Unit, Nairobi, Kenya; 7 Division of Global Health Protection, United States Centers for Disease Control and Prevention, Nairobi, Kenya; 8 Kenya Ministry of Agriculture, Livestock and Fisheries, Nairobi, Kenya; 9 Institute of Tropical Medicine, Antwerp, Belgium

**Keywords:** Clustering, Livestock, Anthrax, Agro-ecological zones, Kenya

## Abstract

**Background:**

Developing disease risk maps for priority endemic and episodic diseases is becoming increasingly important for more effective disease management, particularly in resource limited countries. For endemic and easily diagnosed diseases such as anthrax, using historical data to identify hotspots and start to define ecological risk factors of its occurrence is a plausible approach. Using 666 livestock anthrax events reported in Kenya over 60 years (1957–2017), we determined the temporal and spatial patterns of the disease as a step towards identifying and characterizing anthrax hotspots in the region.

**Methods:**

Data were initially aggregated by administrative unit and later analyzed by agro-ecological zones (AEZ) to reveal anthrax spatio-temporal trends and patterns. Variations in the occurrence of anthrax events were estimated by fitting Poisson generalized linear mixed-effects models to the data with AEZs and calendar months as fixed effects and sub-counties as random effects.

**Results:**

The country reported approximately 10 anthrax events annually, with the number increasing to as many as 50 annually by the year 2005. Spatial classification of the events in eight counties that reported the highest numbers revealed spatial clustering in certain administrative sub-counties, with 12% of the sub-counties responsible for over 30% of anthrax events, whereas 36% did not report any anthrax disease over the 60-year period. When segregated by AEZs, there was significantly greater risk of anthrax disease occurring in agro-alpine, high, and medium potential AEZs when compared to the agriculturally low potential arid and semi-arid AEZs of the country (*p* < 0.05). Interestingly, cattle were > 10 times more likely to be infected by *B. anthracis* than sheep, goats, or camels. There was lower risk of anthrax events in August (*P* = 0.034) and December (*P* = 0.061), months that follow long and short rain periods, respectively.

**Conclusion:**

Taken together, these findings suggest existence of certain geographic, ecological, and demographic risk factors that promote *B. anthracis* persistence and trasmission in the disease hotspots.

## Background

Anthrax is an acute, often fatal disease of animals caused by a soil-borne *Bacillus anthracis* (*B. anthracis*) that exists in two forms; the vegetative toxin-producing form, and the dormant spore form that persists in soil for long periods [1, 2]. The disease is endemic in Africa and Asia where domestic and wild herbivores are infected by ingesting or inhaling spores during feeding and in watering points, whereas humans are infected through contact with infected animal carcasses or their products [2–6]. Cutaneous anthrax is the most common form in humans accounting for 95% of cases [3, 7, 8]. Whereas anthrax has a worldwide distribution, incidence has declined in most developed countries due to livestock vaccination programs and other sanitary measures.

The global incidence of anthrax varies with time and location, triggered by a combination of geographic and ecological factors that support *B. anthracis* persistence, and the changing animal and human demographics that enhance exposure to the pathogen [9–11]. The World Health Organization (WHO) estimates that > 90% of human *B. anthracis* infections globally are attributed to contact with or consumption of livestock and their products and approximately 5% attributed to contact with wildlife [12].

In Kenya, a survey conducted in 2007 reported a human anthrax seroprevalence of 11.3%, with seropositive cases detected in all regions of the country, but ranging from 2% in the north to 27.5% in western regions of the country [13]. The Kenya Directorate of Veterinary Services (KDVS) reports more than 10 livestock anthrax events annually that involve humans, and many more livestock anthrax events that do not involve humans [14]. Wildlife anthrax events are reported in over 30% of Kenyan national conservancies, with > 65% of them reported during the dry seasons and primarily affecting herbivore species: buffaloes, endangered black and white rhinos and elephants that account for > 50% of the affected species and attracting profound conservation interests [6]. Anthrax is among the top priority zoonotic diseases in Kenya and, therefore, the government is developing a risk map for the disease to inform prevention and control measures [15]. Here, we reviewed anthrax records among livestock over the past 60 years in order to describe the temporal and spatial patterns of the disease as a step towards identifying ecological zones with high burden of the disease with the ultimate objective of risk mapping to inform anthrax surveillance, management and control.

## Methods

### Sources of data

We collected data on anthrax events between 1957 and 2017 from three sources. First, the disease events database maintained at Veterinary Epidemiology and Economics Unit (VEEU) of the KDVS headquarters in Kabete, Nairobi, provided data for the 2009–2017 period. Second, the livestock disease event records in the former eight provincial headquarters archives provided additional data for the 1957–2009 period. In 2013, the provinces were dissolved, and the country devolved into 47 semi-autonomous counties. Therefore, we also reviewed disease event records at County Departments of Veterinary Services to obtain data for the 2013–2017 period. From the three data sources, we reviewed standard field disease surveillance report forms referred to as Notifiable Disease-1 form, quarantine records, annual reports, and standard laboratory forms.

### Data collection and analysis

Any livestock death classified as anthrax through clinical or laboratory diagnosis was considered an anthrax event. Using standard guidelines issued by the Kenya’s Directorate of Veterinary Services, a clinical diagnosis was made by animal health practitioners across the country as an acute cattle, sheep or goat disease characterized by sudden death with or without bleeding from natural orifices accompanied by absence of rigor mortis. Further, if the carcass was accidentally opened, failure of blood to clot and/or the presence of splenomegaly were included. In pigs, symptoms included swelling of the face and neck with oedema [16]. A laboratory confirmed anthrax event was diagnosed using Gram and methylene blue stains followed by identification of the capsule and typical rod-shaped *B. anthracis* in clinical specimens submitted to the central or regional veterinary investigation laboratories in Kenya [17].

Using a standard Microsoft Excel® spreadsheet, we captured location of the anthrax events (administrative locations at sub-county level, and Global Positioning System (GPS) coordinates if available), number and species of animals affected, human involvement, event timelines, and clinical and laboratory diagnosis. Subsequently, we cleaned the data to remove duplication and incomplete entries, resulting in final number of 666 anthrax events for analysis. Using the pivot table function within Microsoft Excel®, charts of anthrax event trends were drawn, a frequency distribution prepared, and the data used to map the distribution by counties using Quantum Global Information System (QGIS) version 3.4.4 software (https://qgis.org). We used the livestock census data of 2009 to provide denominators for species comparisons where necessary.

### Spatial clustering of anthrax events

To determine whether anthrax events occurred more frequently in certain geographic location, we used data from 8 counties that reported the highest number of anthrax events, aggregated the events by sub-county, and expressed this as a percent of the total number of the events in the county. We used percentages instead of raw count of anthrax events in order to have a metric for standardization across sub-counties and counties. Each county consisted of 4–12 sub-counties. Four intervals of anthrax events occurrence were used to classify sub-counties in each county; no occurrence (0%), low (1–15%), medium (16–30%), and high (> 30%) occurrences. Using this classification, choropleth maps for each county were developed in QGIS version 3.4.4 software (https://qgis.org), using different color codes to denote no risk (white), low (beige), medium (brown), and high (maroon) risk of anthrax occurrence [18].

To describe spatial clustering by county, we used 86 anthrax events that had GPS coordinates by assigning each to AEZ and fitting a univariate generalized linear mixed-effects model with the Poisson distribution and log link function. Administratively, Kenya has 47 counties while agriculturally it is divided into seven AEZs based on soil types, landforms, and climatic condition that determine agricultural potential. For ease of analysis, the AEZs were reclassified into 5; (i) agro-alpine, (ii) high and medium potential, (iii) semi arid, (iv) arid, and (v) very arid and desert conditions. For the Poisson mixed effects regression, the AEZ, analyzed as a categorical variable was used as fixed effects and subcounty as random effect outcome variable focusing on the expected number of anthrax events in each AEZ as our outcome variable [19]. For AEZs, we used log transformation (ln) of the livestock population in each AEZ as an offset to account for differences in population sizes and then used STATA software (version 14) to fit a random intercept model accounting for spatial dependency.

### Seasonal trends and overall spatio-temporal analyses of anthrax events

**Fig. 1:**
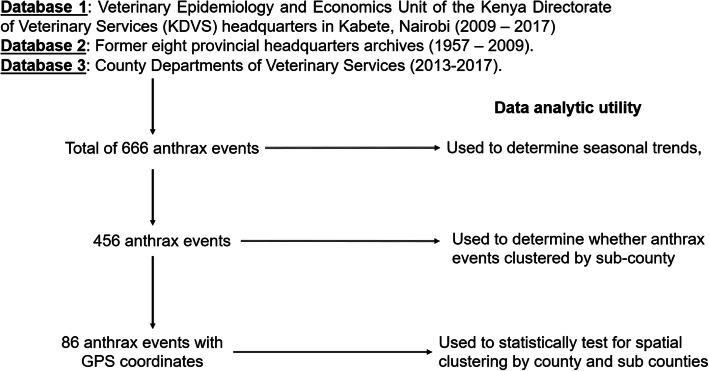
Flow chart illustrating the subsets of data employed in the different levels of the analyses

To determine seasonal trends, all 666 historical anthrax events were aggregated over four seasons experienced in the region; dry and hot season (January to March), wet and cool (April to June), dry and cool (July to September), and wet and hot (October to December). Using percentage of anthrax events, four intervals were created to define counties of no occurrence (0%), low (< 7%), medium (7–10%), and high (> 10%) occurrences. Using time series analysis (R software 3.6.3; https://r-forge.r-project.org/scm/viewvc.php/pkg/timeSeries/?root=rmetrics), we evaluated periodic component covering the defined seasons between 1957 to 2017 [20]. We further analyzed intra-annual variation in the occurrence of the 86 anthrax events using univariate Poisson generalized linear mixed-effects model using principles applied in the above described AEZ model. Here, the calendar month, analyzed as a categorical variable, was used as fixed effects and subcounty as random effect outcome. Figure 1 illustrates the subsets of data employed in the different levels of the analyses. We assessed the overall fit of the models using the chi-square goodness-of-fit tests through computing the sum of the squared deviance and Pearson residuals.

## Results

### Spatial distribution of livestock anthrax events in Kenya

**Fig. 2:**
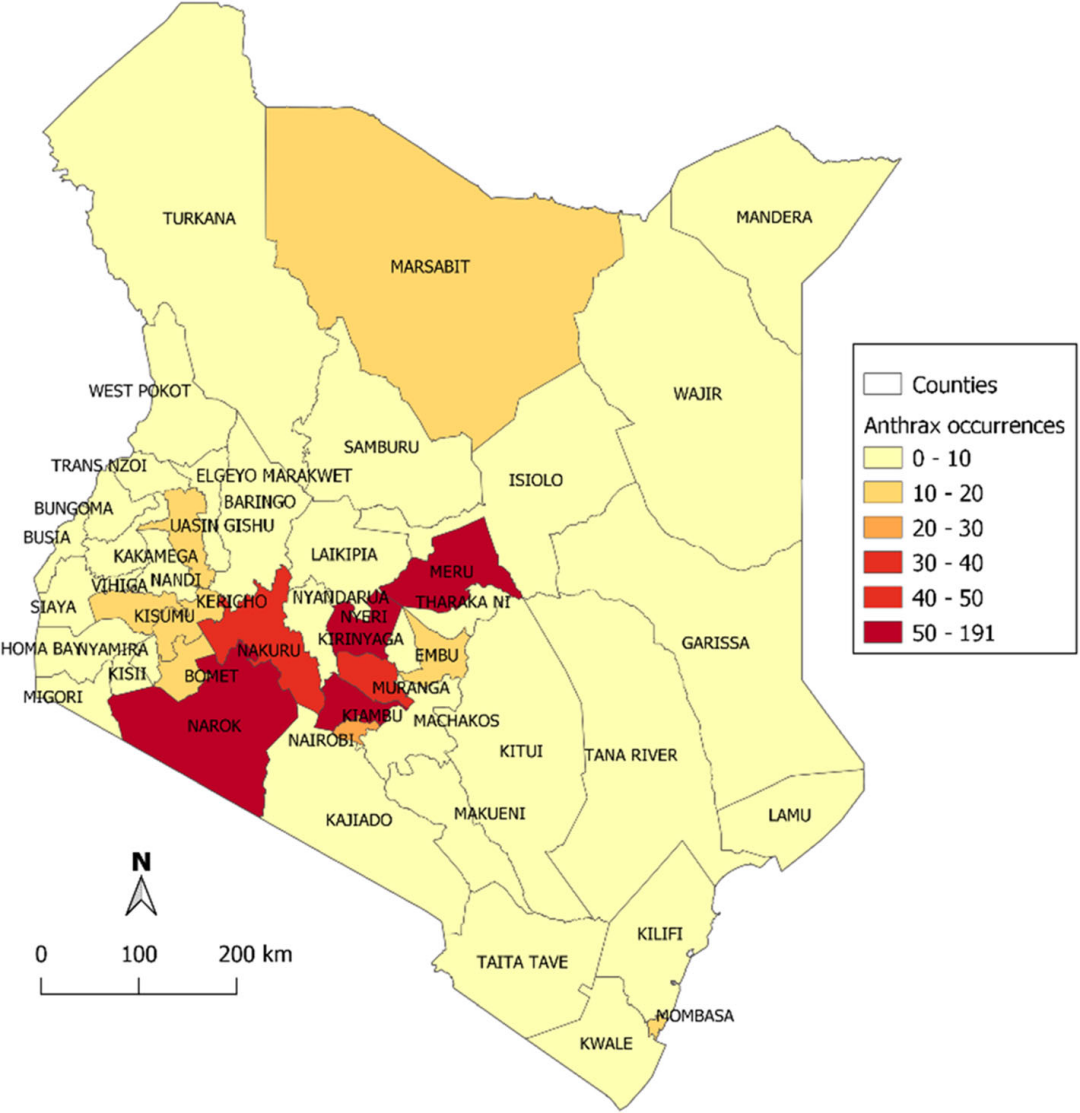
Spatial distribution of anthrax events in livestock by counties, Kenya 1957–2017

In the 60-year period (1957 to 2017), 666 livestock anthrax events in 46 of the 47 (97.9%) counties were identified, of which 17.8% were laboratory confirmed. Only Turkana County located in the far northwest corner of the country did not report an anthrax event. Eight (17.0%) counties reported > 20 events each, most of them located in the south-western and south-central regions of the country including Kiambu county that reported 191 events, Meru, Narok and Nyeri counties that reported 50–60 events, and Murang’a, Nakuru and Nairobi that reported 25–40 events (Fig. 2).

A total of 456 of the 666 (68.5%) anthrax events had sub-county information, and 163 (77.6%) of the 210 sub-counties in Kenya reported at least one anthrax event since 1957. The highest reporting sub-counties and counties were located in Thika (*n* = 32) in Kiambu County, Keyian (*n* = 16) in Narok County, Kikuyu (*n* = 15) in Kiambu County, Rongai (*n* = 13) in Nakuru County, Olulunga (*n* = 13) in Narok County, Kabete (*n* = 12) in Kiambu County, and Sotik (*n* = 12) in Bomet County.

### Anthrax events trend and seasonality

**Fig. 3:**
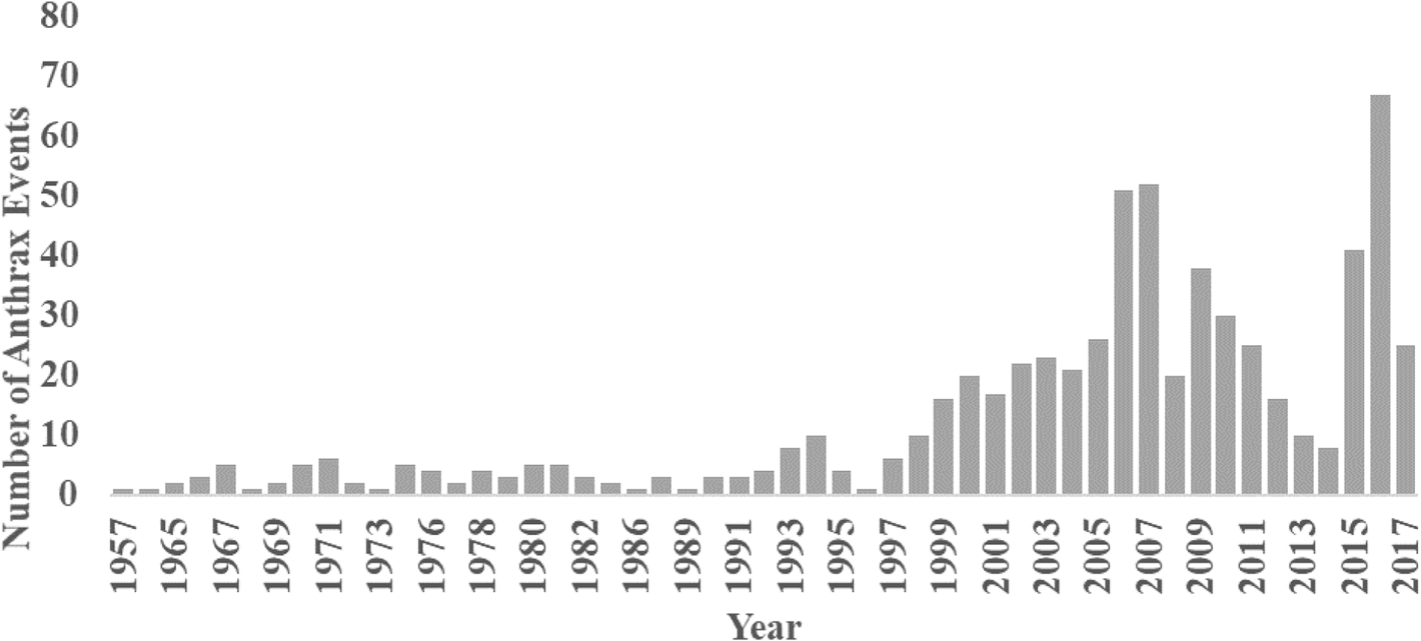
Trend in reported anthrax events in livestock in Kenya, 1957–2017

Between 1957 and 1996, fewer than 10 livestock anthrax events were reported annually (Fig. 3). Thereafter, the number grew steadily, reaching a peak between 2005 and 2007 period when > 50 anthrax events were reported annually. There was a decrease in reported events during the 2011–2014 period, attributed to change in government structure to devolved counties (Fig. 3).

**Fig. 4:**
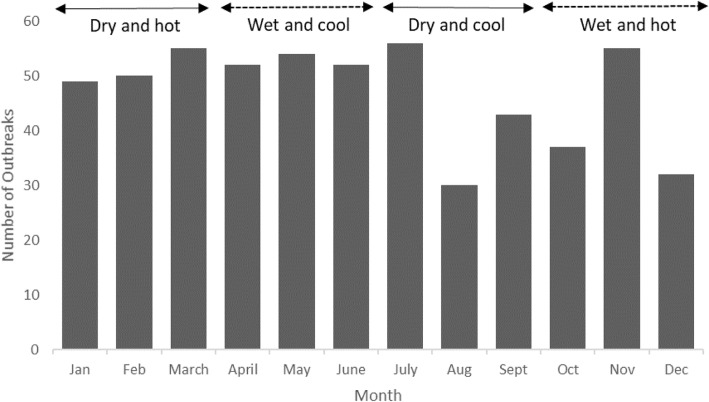
Occurrence of anthrax events aggregated by month and season among Kenyan livestock, 1957–2017

As shown in Fig. 4, descriptive time series analysis revealed less seasonal variation in occurrence of anthrax events. The highest number of anthrax events were reported during the hot and dry January to March season (trend factor = 0.25) while the October to December hot and wet season reported the lowest (trend factor = − 0.26). The wet and cool (April to June) yielded a trend factor of 0.22 while that of dry and cool (July to September) was − 0.2.

### Involvement of livestock species

**Fig. 5:**
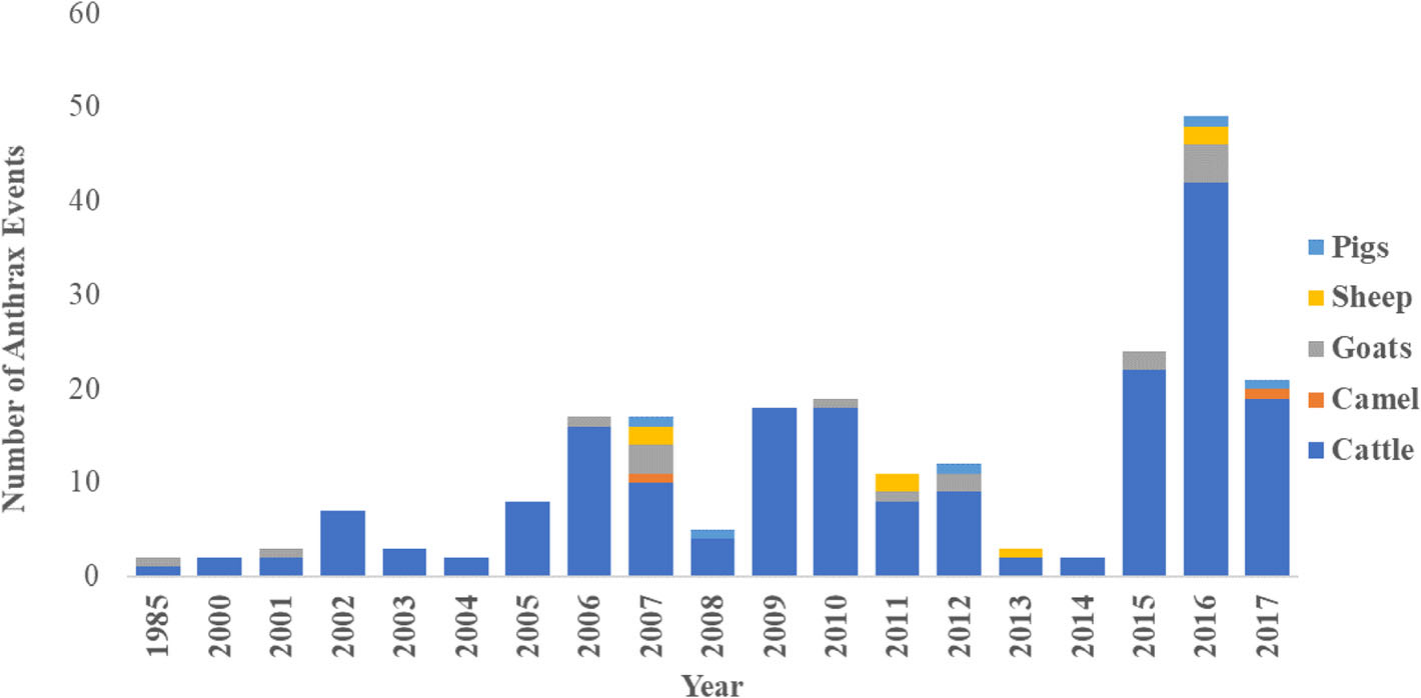
Annual reported numbers anthrax events by livestock species between 1985 and 2017

Based on the 2009 census data, Kenya had 64 million livestock, including goats (27 million), cattle (17 million), sheep (17 million), camels (3 million) and pigs (300 thousand) [21]. However, anthrax associated deaths were more frequently reported in cattle than other species, a trend that was maintained across all years (Fig. 5). The ratio of cattle to small ruminant reported anthrax events was between 10:1 and 20:1.

### Spatial clustering

**Fig. 6:**
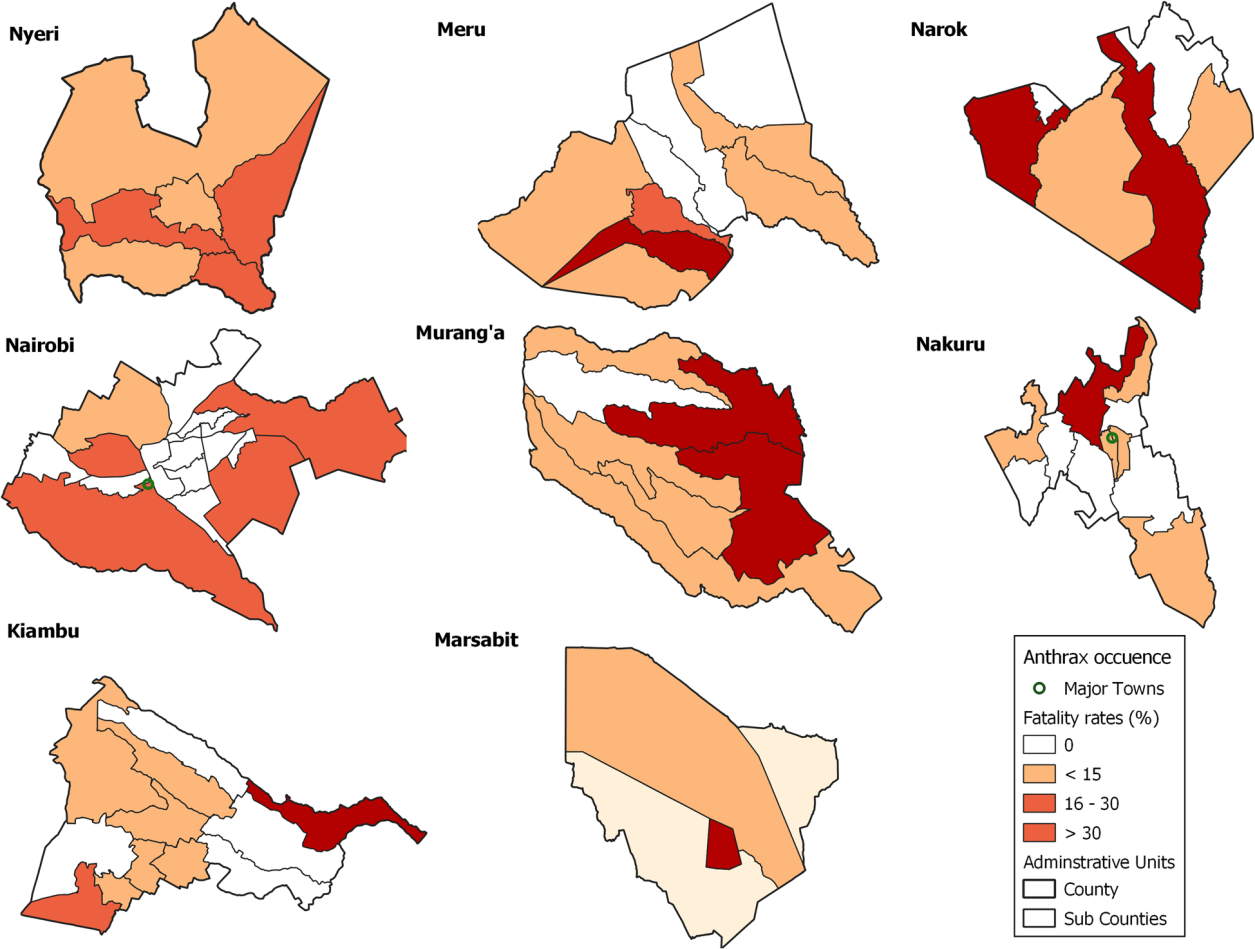
Density map for anthrax events in livestock in eight Kenyan counties that reported the highest number of anthrax events

We investigated spatial distribution of anthrax events in eight counties that reported 71.3% (475/666) of the livestock anthrax events, aggregated into sub-counties. The counties were Kiambu (*n* = 191 events), Meru (*n* = 56), Nyeri (*n* = 53), Narok (*n* = 51), Murang’a (*n* = 39), Nakuru (*n* = 37), Nairobi (*n* = 28), and Marsabit (*n* = 20). Of the 69 sub-counties in these counties, 25 (36.2%) reported zero events, whereas 8 (11.6%) reported > 30% (high risk) anthrax events (Fig. 5). Another 12 (17.4%) sub-counties reported 16–30% (medium risk) events whereas 24 (34.8%) sub-counties reported 1–15% (low risk) events (Fig. 6). This clustering trend was maintained over time as demonstrated by stratification of the data into 20-year periods; 1957–1977, 1978–1997, and 1998–2017. The 20-year period was chosen because it provided the minimum number of observations for adequate and meaningful analyses. Our data showed a larger proportion of anthrax events reported in dry seasons relative to wet seasons, regardless of region of the country or seasonal temperature variations.

**Fig. 7:**
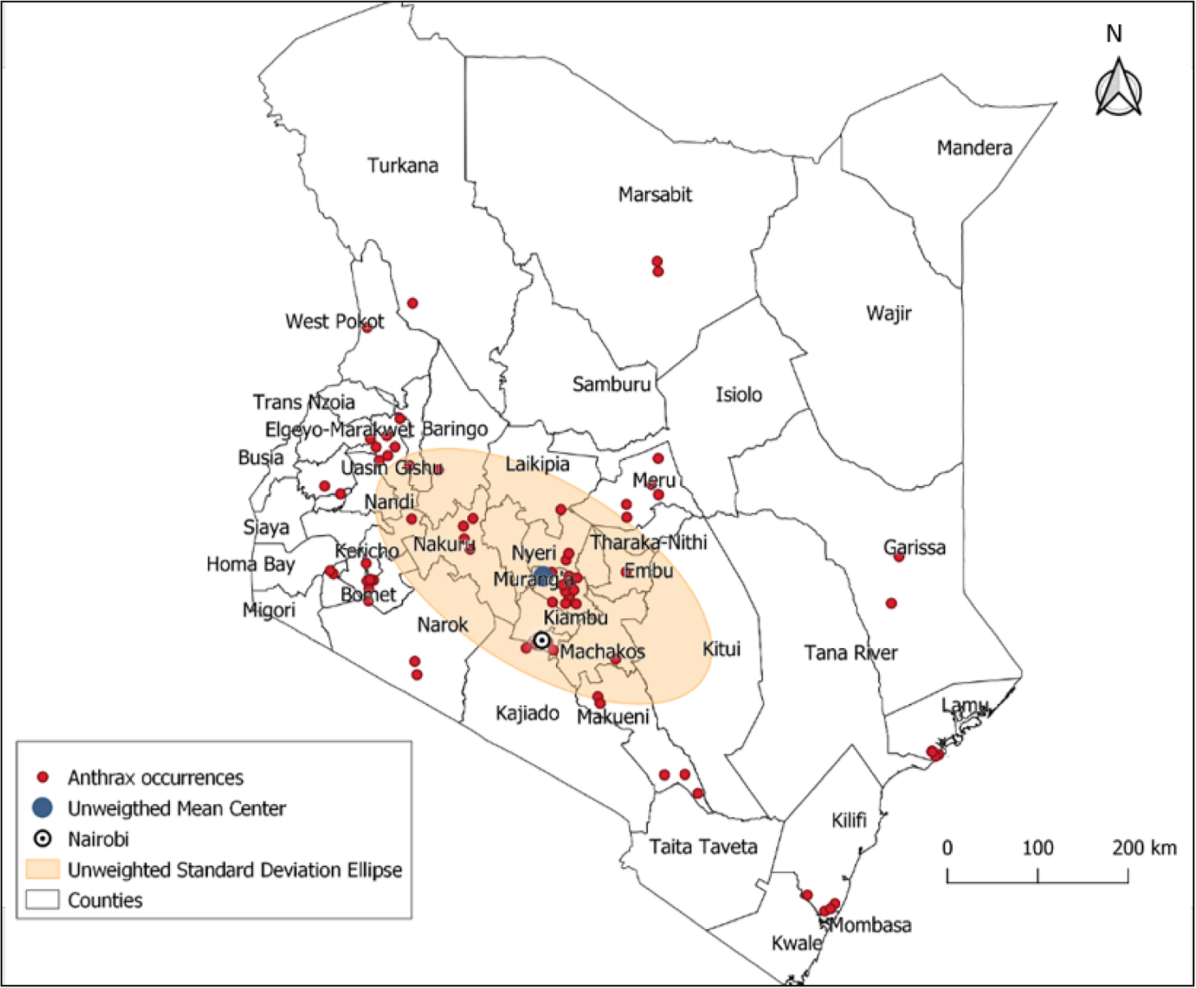
Spatial distribution of the 86 anthrax events with GPS coordinates

Analysis of 86 anthrax events with GPS coordinates (Fig. 7), showed progressive decrease in the number of events from the agro-alpine (highest), high and medium potential, semi-arid, arid, to very arid zones (lowest) as shown in Table 1. The High and Medium potential was used as the referent category since it constituted the largest proportion of events (68.5%). The likelihood ratio test conducted during the analyses showed that inclusion of sub-county random effect provided a substantially better fit than the standard Poisson regression (*P* = 0.000), supporting clustering at the sub-county level (Table 1). Using the 86 anthrax events, the data shows significant differences in number of events associated with calendar month using likelihood test ratio (*P* = 0.02, Table 2). For any given county, there was lower risk of anthrax events in August (*P* = 0.034) and December (*P* = 0.061), months preceded by long and short rain periods, respectively (Table 2). In assessing the fit of the models, the deviance statistic had a *P* = 0.90 whereas the Pearson statistic had *P* = 0.27 suggesting a good model fit.

**Table 1:** Assessment for spatial clustering of anthrax events using the Poisson mixed effects regression model with agro-ecological zone as fixed effect

Variable	Variable category	Incidence rate ratio	Coefficient 95% confidence interval	***P*** value
Agro-ecological zone	High and Medium potential	Reference		
	Agro-alpine	1.23	[0.82, 1.84]	0.32
	Semi-arid	0.90	[0.52, 1.57]	0.71
	Arid	0.48	[0.23, 1.00]	0.05
	Very Arid	0.39	[0.23, 0.67]	< 0.001
Random effects Subcounty identification:	–	0.72		–

Llikelihood ratio test versus Poisson regression chibar2(01) = 536.87; Prob> = chibar2 = 0.000

**Table 2:** Assessment of temporal trends of anthrax events using Poisson mixed-effects regression model with calendar month as fixed effect across years

Variable	Variable category	Coefficient	95% confidence interval	***P*** value
Month	January	Reference		
	February	1.04	[0.70, 1.63]	0.43
	March	1.11	[0.77, 1.65]	0.55
	April	1.05	[0.72, 1.55]	0.76
	May	1.09	[0.76, 1.62]	0.62
	June	1.05	[0.72, 1.55]	0.76
	July	1.13	[0.79, 1.67]	0.49
	August	0.61	[0.39, 0.97]	0.03
	September	0.88	[0.58, 1.31]	0.53
	October	1.32	[0.50, 1.15]	0.19
	November	1.11	[0.77, 1.65]	0.55
	December	0.66	[1.65, 1.01]	0.06
Random effects County identification:	–	Standard deviation =1.57		–

Likelihood ratio test versus Poisson regression chibar2(01) = 1441.2; Prob> = chibar2 = 0.0000

## Discussion

This study documents a relatively high frequency (mean = 11 per year) and widespread distribution (95% of the counties) of anthrax events among livestock in Kenya over 60 years, a trend similar to that reported among wildlife in the country [6]. Given that the data were derived from a passive surveillance system with significant underreporting, the actual frequency may be be two- to three-fold higher [22–25]. The anthrax events increased in recent years (since 1997), probably associated with enhanced disease surveillance and reporting by the country. The increased disease reporting is due to growing awareness of importance of timely disease detection as a measure of preparedness and response for emerging infectious diseases by the Government of Kenya, and compliance with the global animal and human health agencies such as the World Organization for Animal Health (OIE) and WHO. Similar reporting trends have been documented in other infectious diseases including influenza, viral hemorrhagic fevers, rabies and brucellosis [26–29]. A higher number of anthrax events was observed in the southwestern region of the country, possibly attributed to more developed infrastructure that promotes disease reporting, whereas the low reporting rates in arid and semi-arid marginalized northern regions of the country are likely due to poor infrastructure and access to animal health services [25].

More granular analysis at sub-county and AEZ levels showed clustering of anthrax disease events at certain locations, likely associated with the existence of geographic, ecological, and demographic risk factors that promote *B. anthracis* persistence and transmission. For instance, within eight high burden counties, 36% of the sub-counties did not report anthrax disease, whereas 11% were responsible for > 30% of anthrax events. When analyzed by AEZ, there was significantly higher occurrence of disease in agriculturally productive zones (agro-alpine, high potential, medium potential) when compared to the dry, less productive lands (semi-arid, arid, very arid). These findings are in agreement with a study in Mongolia that reported fewer anthrax cases in the arid and semi-arid areas of the country [30]. While the association between geographic factors and persistence of *B. anthracis* has been documented, the preferred AEZ appears to vary with on the global geo-climatic oscillations [9, 31].

The anthrax disease events were less variable when analyzed by season, even though more occurred in dry than wet seasons. We reported less anthrax events in August and December across years and counties, months that a follow the occurrence of heavy rains. This trend closely mirrored that of other studies including a wildlife study in Kenya that reported higher frequency of anthrax events in the dry season [6, 10, 32–34]. Of geographic factors, studies demonstrate strong correlation between rainfall and temperature with *B. anthracis* persistence and transmission, while there is less agreement on the role of soil, vegetation, population density, and other factors [9, 11, 35].

An interesting finding in our study was the 10-fold higher anthrax events in cattle when compared to other livestock, findings similar to those reported in Australia, Lesotho, Zambia, and Zimbabwe [36–40]. Whereas it is possible that this finding represents reporting bias in favor of cattle, it may not fully explain the high levels of cattle events reported since livestock anthrax reports are closely linked with human infections that drive the reporting. It is possible that cattle are more susceptible to anthrax disease, perhaps due to ingesting higher doses of the pathogen because of their grazing niche, or preferential mechanical transmission of *B. anthracis* by insects and biting flies [1, 35]. However, this is not supported by experimental challenge studies, which show sheep and goats as more susceptible than cattle [1]. The importance of the finding that cattle are more heavily affected than other livestock is that an anthrax control program involving routine vaccination of only cattle may be both effective and economically feasible.

Our study had several limitations. The use of historical data from passive surveillance represented a bias associated with higher reporting in more developed regions of the country when compared to the remote, rural, dry regions. In addition, while it was convenient to select the eight counties with the highest burden of anthrax events to investigate clustering in order to ensure sufficient numbers for analysis, this may have further exacerbated the reporting bias. The low number of anthrax events with geocodes precluded niche modeling studies to begin to tease out ecological factors important in disease occurrence. This limitation is addressed in ongoing prospective study in the country.

## Conclusion

Anthrax remains a high priority zoonotic disease in sub-Saharan Africa associated with significant negative impact on public health and livestock productivity. The data presented here provides Kenya and the region with a roadmap for targeting surveillance, and prevention and control measures to certain AEZ, even though additional studies to support these findings are necessary.

## Data Availability

The data that support the findings of this study are available from Directorate of Veterinary Services, State Department of Livestock, Ministry of Agriculture, Livestock and Fisheries, Kenya but restrictions apply to the availability of these data, which were used under license for the current study, and so are not publicly available. Data are however available from the authors upon reasonable request and with permission of Director of Veterinary Services, Kenya.

## Abbreviations

AEZ: Agro-ecological zone
*B. anthracis*: *Bacillus anthracis*
GPS: Global Positioning System
KDVS: Kenya Directorate of Veterinary Services
OIE: World Organization for Animal Health (OIE)
QGIS: Quantum Geographic Information System
VEEU: Veterinary Epidemiology and Economics Unit
WHO: World Health Organization
